# Carbon Nanofibers Have IgE Adjuvant Capacity but Are Less Potent Than Nanotubes in Promoting Allergic Airway Responses

**DOI:** 10.1155/2013/476010

**Published:** 2013-08-19

**Authors:** Unni Cecilie Nygaard, Mari Samuelsen, Calin Daniel Marioara, Martinus Løvik

**Affiliations:** ^1^Division of Environmental Medicine, Norwegian Institute of Public Health, P.O. Box 4404 Nydalen, 0403 Oslo, Norway; ^2^SINTEF Materials and Chemistry, Høyskoleringen 5, 7465 Trondheim, Norway; ^3^Department of Cancer Research and Molecular Medicine, Faculty of Medicine, Norwegian University of Science and Technology, Olav Kyrres Gate 9, 7489 Trondheim, Norway

## Abstract

There is a growing concern for the possible health impact of nanoparticles. The main objective of this study was to investigate the allergy-promoting capacity of four different carbon nanofiber (CNF) samples in an injection and an airway mouse model of allergy. Secondly, the potency of the CNF was compared to the previously reported allergy-promoting capacity of carbon nanotubes (CNT) in the airway model. Ultrafine carbon black particles (ufCBP) were used as a positive control. Particles were given together with the allergen ovalbumin (OVA) either by subcutaneous injection into the footpad or intranasally to BALB/cA mice. After allergen booster, OVA-specific IgE, IgG1, and IgG2a in serum were measured. In the airway model, inflammation was determined as influx of inflammatory cells (eosinophils, neutrophils, lymphocytes, and macrophages) and by mediators (MCP-1 and TNF-*α* present in bronchoalveolar fluid (BALF)). CNF and CNT both increased OVA-specific IgE levels in the two models, but in the airway model, the CNT gave a significantly stronger IgE response than the CNF. Furthermore, the CNT and not the CNF promoted eosinophil lung inflammation. Our data therefore suggest that nanotube-associated properties are particularly potent in promoting allergic responses.

## 1. Introduction

Allergic airway diseases are characterized by eosinophil and lymphocyte lung inflammation, as well as allergen-specific IgE in serum. Ultrafine particles present in ambient air have, in animal models and in humans, been demonstrated to modulate airway inflammations and promote allergic responses in the lung [1–4]. Carbon nanotubes (CNT) are particles applied in an increasing number of consumer products and are also incidental components in indoor air pollution [5, 6]. Carbon nanofibers (CNF) are useful in many of the same applications as CNT, such as Li-ion batteries and polymer nanocomposites [7]. In parallel to the increasing manufacture of CNT and CNF, there is a growing concern for the health impact of these nanoparticles in occupational workers and consumers in general, also in relation to allergy. Inhalation studies in rodents suggest that CNT may induce toxic effects like transient inflammation, fibrosis, and granuloma formation [8], and mice with a preexisting allergic inflammation have been reported to be particularly susceptible [9]. Further, single-walled (sw) and multiwalled (mw) CNT have been reported to increase allergen-specific IgE levels, eosinophil airway inflammation, and Th2-associated cytokine responses in mice models [2, 10, 11]. In support, Park and coworkers demonstrated increased total IgE, Th2-associated cytokine, and B cell levels in mice after a single intratracheal exposure to mwCNT [12]. However, little is known about the characteristics of CNT responsible for the allergy-promoting effect. The possible allergy-promoting properties of CNF have not been investigated.

The main objective of this study was to investigate the allergy-promoting capacity of four different CNF samples in an injection and an airway mouse model of allergy. Secondly, the potency of the CNF was compared to the previously reported allergy-promoting capacity of CNT and ultrafine carbon black particles (ufCBP) in the airway model. Knowledge on which physicochemical characteristics of the particles are important for their biological effects is essential to enable manufacturing of particles with low toxicity. We, therefore, explored the importance of major physicochemical properties of carbon nanoparticles on their capacity to modulate allergy, by comparing the allergy-promoting capacity of samples having different physicochemical properties. Four samples of well-characterized, qualitatively different CNF produced in the same pilot plant were tested in both mouse models (CNF A, B, C, and D [7]). By deliberate use of different conditions and purification steps during the CNF manufacturing process, four samples with different particle properties such as fiber width and length, open versus closed channels, metal impurity content, relative surface area, presence of structural defects, and relative amount of fibers to other graphitic material were produced. Samples of swCNT, mwCNT, and “spherical” ufCBP (Printex90), previously shown to promote allergic responses in the two mouse models used [2], were also included in the airway model. The allergy parameters allergen-specific IgE levels in serum and eosinophil lung inflammation were measured. Allergen-specific IgG2a antibodies and the presence of inflammatory cells, MCP-1, and TNF-*α* in bronchoalveolar lavage fluid (BALF) were also determined, to reflect Th1- and general inflammatory responses.

## 2. Material and Methods

### 2.1. Animals

Female inbred BALB/cAnNCrl mice (Charles River, Sulzfeld, Germany) were 6-7 weeks old upon arrival and were acclimatised for one week. Four animals were housed per cage in Innorack IVC (Innovive Inc., CA, USA), containing Nestpaks filled with aspen 4HK bedding (Datesand Ltd, Manchester, UK). The mice were exposed to a 12 hr/12 hr light/dark cycle (30–60 lux in cages), regulated room temperature (20  ±  2°C), and 40–60% relative humidity. Pelleted food (RM1, SDS, Essex, UK) and tap water were provided *ad libitum*. The experiments were performed in conformity with the laws and regulations for experiments with live animals in Norway and were approved by the Norwegian Experimental Animal Board under the Ministry of Agriculture (FOTS ID numbers 678 and 1005).

### 2.2. Particle Source and Characteristics

Four qualitatively different batches of carbon nanofibers (CNF A, B, C and D) were kindly provided by Statoil and Elkem Carbon AS (Kristiansand, Norway). The particles were produced from natural gas in a catalytic chemical vapor deposition (CVD) process and processed as reported [11]. The swCNT and mwCNT, also produced by CVD method, were obtained from Sigma-Aldrich (St. Louis, MO, USA, Cat. numbers 636797 and 636487, resp.). Ultrafine carbon black particles (ufCBP, Printex90), kindly provided by Degussa (Köln, Germany), were included as a positive reference particle for adjuvant activity in mice [1, 2]. All particle samples were characterized by transmission electron microscopy (TEM), and the specific surface area was determined by nitrogen adsorption using the Brunauer, Emmet, and Teller method (BET). The endotoxin levels were determined in particle supernatants by the limulus amebocyte lysate (LAL) assay as previously described [2, 7], and the pH of the particle supernatants was measured (Mettler Toledo AS, NY, USA).

### 2.3. Particle and Allergen Preparations

Ovalbumin (OVA, Gal d1; chicken egg albumin, grade VII, Sigma, St. Louis, MO, USA) was used as allergen after removal of endotoxin by Detoxi-Gel Endotoxin Removing Gel (Pierce, Rockford, IL, USA). The final endotoxin level measured less than 0.025 ng/mg OVA, as determined by the LAL assay. Particle suspensions were prepared in Hank's balanced salt solution (HBSS), with BALB/cA mouse serum to facilitate suspension of particles, and microtip probe sonicated as described in Nygaard et al. [2]. The CNT and CNF were even then mainly observed as agglomerates in TEM.

### 2.4. Injection Model

The IgE adjuvant capacity of the CNF particles was initially investigated in a footpad injection model suitable for studying respiratory allergy adjuvants (method reviewed in [13], performed as described in [2]). In short, groups of eight mice were given a single dose of 200 *µ*g of CNF A, B, C, D, or ufCBP together with 10 *µ*g OVA, OVA boosted (10 *µ*g) after 21 days and terminated on day 26. Sera were collected and stored at −20°C until quantification of OVA-specific IgE, IgG1, and IgG2a by ELISA (see below). A particle dose of 200 *µ*g was chosen, a dose previously shown to give a pronounced adjuvant effect of CNT in this injection model [2].

### 2.5. Intranasal Model

The adjuvant capacity of the particles after airway exposure was examined in an intranasal mouse model. Exposures were performed as described in [2], and a particle dose previously giving pronounced effects of CNT in the intranasal model was chosen [2]. In short, 133.3 *µ*g CNF A, B, C, D, swCNT, mwCNT, or ufCBP together with 10 *µ*g OVA was given intranasally to groups of ten mice on three consecutive days, giving a total dose of 400 *µ*g particles and 30 *µ*g OVA during sensitization. All mice were OVA-boosted intranasally (10 *µ*g per day) on days 21, 22, and 23. On day 26, the animals were deeply anesthetized by a 0.3 mL intraperitoneal injection of a mixture of Zoletil forte (17 mg/kg tiletamine and 17 mg/kg zolazepam; Virbac International, Carros Cedex, France) and Narcoxyl (13.6 mg/kg xylazine; Intervet/Schering-Plough Animal Health, Boxmeer, The Netherlands). Sera were stored at −20°C until quantification of OVA-specific IgE, IgG1, and IgG2a by ELISA (see below). Lung inflammation was determined by total and differential cell counts and cytokine levels in BALF (see below). Based on previous findings with sw and mw CNT [2], the cytokines TNF-*α* and MCP-1 in BALF were selected.

### 2.6. Detection of IgE, IgG1, and IgG2a Anti-OVA Antibodies

Detection of OVA-specific IgE, IgG1, and IgG2a antibodies was performed by ELISAs as described in Nygaard et al. [2]. The sera to be tested were diluted 1 : 10 for IgE and IgG2a, and for IgG1 diluted 1 : 4 000 and 1 : 20 000 for the injection and intranasal models, respectively. Values outside the dynamic range of the standard curve were set to a value just below/above the detection limit.

### 2.7. BALF Collection, Preparation, and Analyses

BALF was collected and prepared, and total and differential cell counts were determined as previously described [2, 14]. In short, the supernatant from a first lavage was stored at −80°C until cytokine measurements, while the cells from all three lavages were pooled, counted, and stained, and 400 cells per slide were differentiated into neutrophils, eosinophils, epithelial cells, lymphocytes, and macrophages. A number of macrophages appeared to contain particles, but since we could not determine whether the particles were taken up or were adsorbed to the cell membranes, they were counted as particle-associated macrophages. In BALF supernatants, the levels of tumor necrosis factor-alpha (TNF-*α*) and monocyte chemoattractant protein-1 (MCP-1) were determined using BD Cytometric Bead Array (CBA) Mouse Soluble Protein Flex Sets, as previously described [2].

### 2.8. Data Analysis

The data were log10-transformed to meet the assumptions for running one-way ANOVAs. In order to determine which groups differed significantly from the others when the ANOVAs were positive (*P* ≤ 0.05), pairwise comparisons were performed by Tukey's post hoc test. Group differences were considered statistically significant if *P* ≤ 0.05. All statistical analyses were performed with SigmaStat Statistical Analysis System for Windows Version 2.03 (Jandel Scientific, Erkrath, Germany).

## 3. Results

### 3.1. Particle Characterization

**Figure 1:**
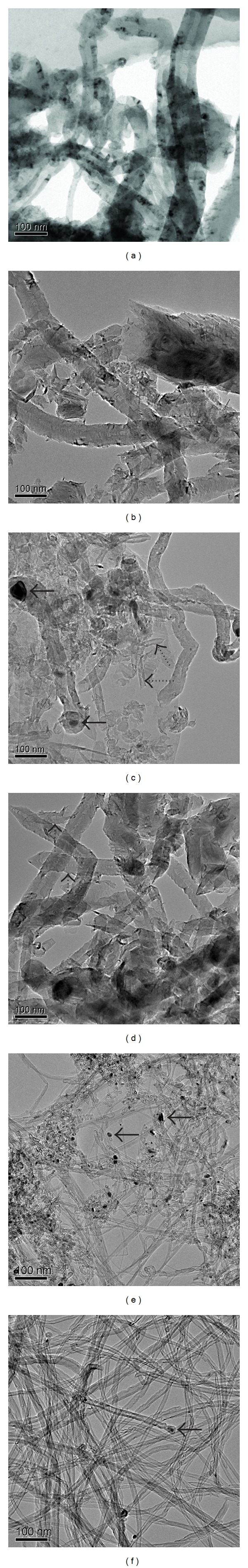
TEM micrographs of the particle samples. Micrographs of CNF A (a), CNF B (b), CNF C (c), CNF D (d), swCNT (e), and mwCNT (f) illustrate the difference in width and length between the CNF and CNT. Examples of metallic particle presence are indicated by dense arrows. Most metallic particles are embedded in the carbon material. Shorter fragments of fibers are indicated by dotted arrows.

Particle sample properties are shown in Table 1, while the methods used for particle characterization and more comprehensive characterization of the samples are previously reported [2, 7]. TEM micrographs at similar magnifications from CNF A, B, C, D, swCNT, and mwCNT samples are presented in Figures 1(a)–1(f), respectively. Systematic inspection of the TEM micrographs revealed that the majority of the CNF agglomerates were within the size range 0.6 *µ*m to 2.6 *µ*m. The CNF were wider than the CNT, and the mwCNT were wider than the swCNT, as illustrated in the micrographs and given by the mean fiber width presented in Table 1. Most CNF are fractured or broken and therefore, on an average, are shorter than the CNT (as illustrated by dotted arrows in Figure 1, Table 1). Inspection of the TEM micrographs also showed that CNF samples and the swCNT sample contain disordered graphitic material, while the mwCNT sample is more homogeneous. High numbers of metal particles were observed in CNF A, CNF C, and swCNT, mainly embedded in the carbon material (indicated by arrows in Figures 1(c), 1(e), and 1(f)). CNF A and CNF C have a higher surface area, a lower mean fiber diameter, and a higher presence of structural defects than CNF B and CNF D samples (Table 1).

**Figure 2:**
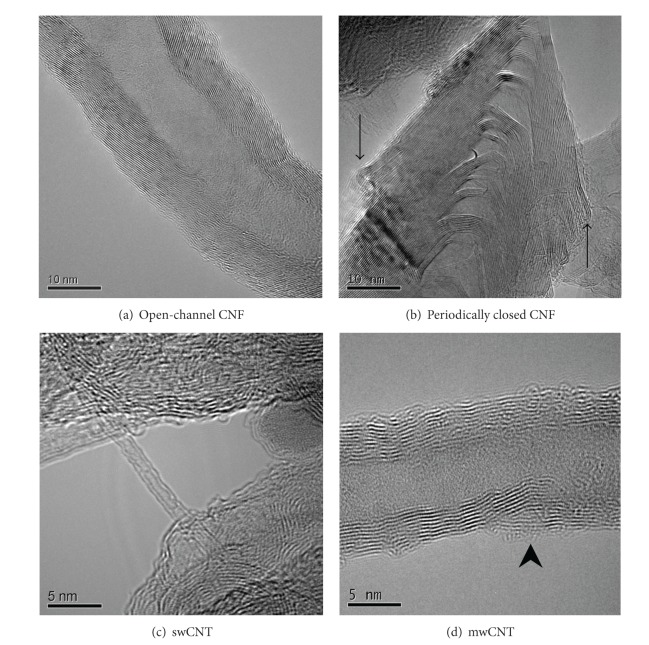
High-resolution images of particles, illustrating their internal structure. The herringbone CNFs consisted of graphite layers arranged at an angle to the axis of the filament structure ((a), (b)). The micrographs show the structure of a typical open-channel fiber found in all CNF samples (a) and a periodically closed fiber present in large amounts only in CNF B and CNF D (b). Many graphite layers in the periodically-closed type of fiber have closed ends at the surface, indicated by arrows in (b). A typical example of a swCNT, together with disordered graphitic material, is shown in (c). An example of a typical defect mwCNT is shown in (d); a defect in the wall is indicated by an arrow head.

Figure 2 shows the typical internal structure of CNF and CNT in high-resolution TEM images. The CNT consist of single or multiple concentric graphene sheets rolled into form of cylinders (Figures 2(c) and 2(d)). In contrast, the CNF were classified into two main types of stacked arrangements of graphite cones: with channels that are mostly open (Figure 2(a)) or with periodically closed channels (Figure 2(b)). The latter are also characterized by closed layers on the CNF surface, as indicated by arrows. Although the open channel type was found in all CNF samples, only the CNF B and CNF D samples contained a high fraction of the periodically closed channel type. As previously reported [2], only half of the tubes in the swCNT sample were identified as single-walled, the other half being multiwalled. Most tubes in the mwCNT sample were defective, as exemplified in Figure 2(d) (arrowhead).

The particles were also studied after preparation in the medium used for the *in vivo* studies. As judged by TEM analyses, the sonication process did not affect the length of the CNT, whereas some breakage of the CNF was observed (data not shown). This was most probably due to a lower mechanical strength of the fibers compared to the tubes, caused by the different stacking of the graphite layers. The acidity (pH) of the particle supernatants did not differ markedly between the particles, and the endotoxin levels were below detection limit for all particles, except swCNT which had a detectable but low level (Table 1).

### 3.2. OVA-Specific IgE, IgG1, and IgG2a Levels in Serum after Coinjection of Particles and OVA in the Footpad

**Figure 3:**
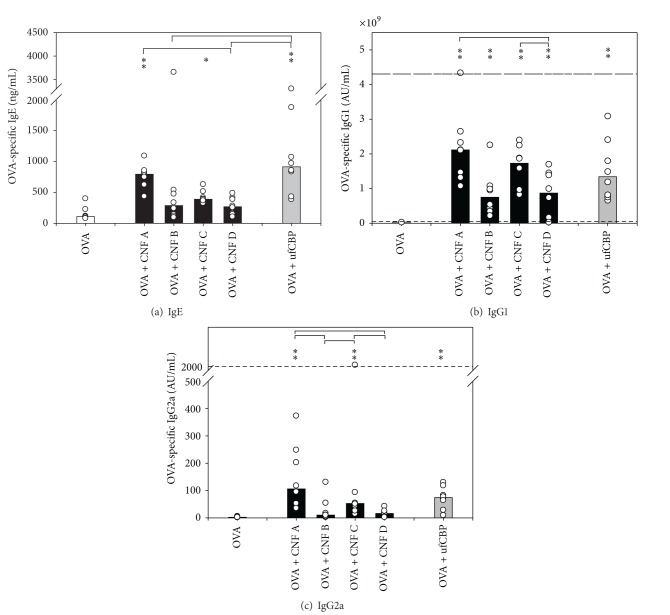
Allergen-specific antibodies after subcutaneous coinjection of allergen and particles. Serum levels of OVA-specific IgE (a), IgG1 (b), and IgG2a (c) 26 days after subcutaneous injection into one hind footpad of 10 *µ*g OVA alone (white bars) or together with 200 *µ*g CNF (samples A, B, C, and D; black bars) or ufCBP (light grey bars). On day 21, all mice were boosted with 10 *µ*g OVA in the footpad. Values (ng or arbitrary units (AU) per mL) for individual mice (circles) and median values (columns) for groups of eight mice are shown. If values were outside the dynamic range of the ELISA assays, the dotted lines indicate the lower or upper quantitative detection limits for the assays. Asterisk denotes significant differences compared to the OVA group, **P < 0.001, *P < 0.05. Brackets denote statistically significant differences between the particle groups, *P* < 0.05.

In the footpad injection model, OVA-specific IgE levels were statistically significantly higher in mice treated with OVA and CNF A, CNF C, or ufCBP than with OVA alone (Figure 3(a)). ufCBP elicited significantly higher IgE levels than CNF B and CNF D, and CNF A elicited significantly higher levels than CNF D. OVA-specific IgG1 levels were significantly elevated by all particles, but CNF A and CNF C had higher IgG1-adjuvant activity than CNF B and D, but statistically significant only for CNF D (Figure 3(b)). CNF A, CNF C, and ufCBP gave weakly but significantly elevated levels of OVA-specific IgG2a levels (Figure 3(c)).

### 3.3. OVA-Specific IgE, IgG1, and IgG2a Levels in Serum after Intranasal Coexposure to Particles and OVA

**Figure 4:**
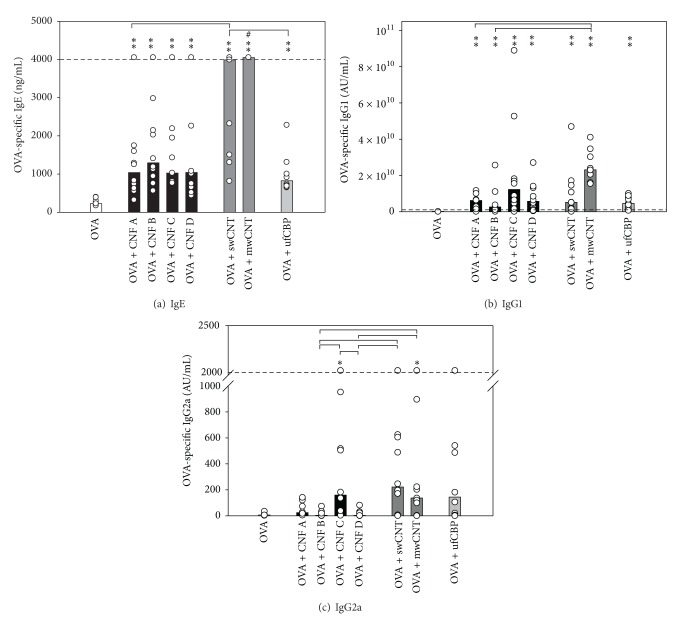
Allergen-specific antibodies after intranasal coexposure to allergen and particles. Serum levels of OVA-specific IgE (a), IgG1 (b), and IgG2a (c) 26 days after intranasal exposure to 10 *µ*g OVA alone (white bars) or together with 133 *µ*g particles on days 0, 1 and 2 (total dose of 30 *µ*g OVA and 400 *µ*g particles). The four samples of CNF (black bars), swCNT and mwCNT (dark grey bars), and ufCBP (light grey bars) were used. On days 21, 22, and 23, all mice were boosted intranasally with 10 *µ*g OVA (per day). Values (ng or arbitrary units (AU) per mL) for individual mice (circles) and median values (columns) for groups of ten mice are shown. If values were outside the dynamic range of the ELISA assays, the dotted lines indicate the lower or upper quantitative detection limits for the assays. Asterisk denotes significant differences compared to the OVA group, **P < 0.001,*P < 0.05. # Denotes significant differences compared to all other particle groups, *P* < 0.05. Brackets denote statistically significant differences between particle groups, *P* < 0.05.

In the intranasal model, all particles significantly increased OVA-specific IgE levels compared to the OVA control group (Figure 4(a)). The four CNF samples and ufCBP elicited similar levels of IgE, while in comparison, the sw and mw CNT elicited very high IgE levels. Also the OVA-specific IgG1 levels were significantly increased by all particles, and mwCNT elicited significantly higher levels than CNF A and CNF B (Figure 4(b)). As compared to the OVA control group, OVA-specific IgG2a levels were significantly increased after exposure to CNF C and mwCNT and also tended to be increased by swCNT (*P* = 0.079) and ufCBP exposure (*P* = 0.088; Figure 4(c)).

### 3.4. Airway Inflammation after Intranasal Coexposure to Particles and OVA

**Figure 5:**
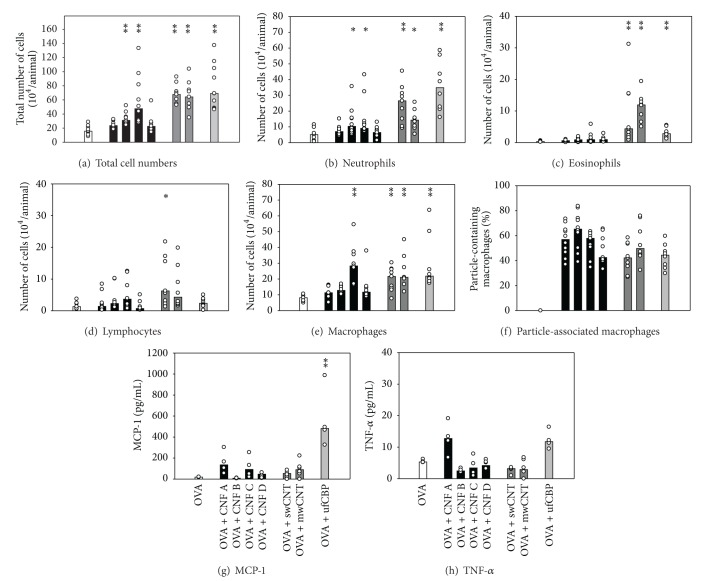
Airway inflammation measured after intranasal coexposure to allergen and particles. Cell numbers and cytokines in BALF collected following OVA booster, 26 days after intranasal exposure of mice to allergen alone or together with particles (as described in legend of Figure 4). The total cell numbers (a) were determined on a Coulter cell counter, whereas the number of neutrophils (b), eosinophils (c), lymphocytes (d), and macrophages (e) and the percent of particle-associated macrophages (f) were determined by counting 400 cells per stained cytoslide. The amount of MCP-1 (g) and TNF-*γ* (h) in BALF supernatants was determined by CBA assay. Values for individual mice (circles) and group medians (columns) for groups of ten mice are shown, except MCP-1 and TNF-*α* where only half of the samples could be measured. Asterisk denotes significant differences compared to the OVA group, **P < 0.001, *P < 0.05. Statistically significant differences between particle groups are given in the results section.

In the intranasally exposed mice, CNF B, CNF C, sw and mw CNT, and ufCBP with OVA significantly increased the total number of BALF cells, compared to the group given OVA alone (Figure 5(a)). The total cell numbers were significantly higher in the groups given swCNT, mwCNT, and ufCBP than in the CNF groups, with the exception of CNF C which elicited significantly higher cell numbers than CNF A and CNF D. The particles that increased the total cell numbers also significantly increased the neutrophil cell numbers (Figure 5(b)). The eosinophil numbers were low in the groups treated with OVA alone or together with the CNF samples. Exposure to OVA with swCNT or mwCNT, however, induced significantly higher eosinophil numbers than OVA alone or OVA with any of the CNF samples (Figure 5(c)). ufCBP with OVA also increased the eosinophil numbers, although the numbers were only significantly higher than in mice treated with OVA alone or together with CNF A and CNF B. The number of lymphocytes was slightly, but statistically significantly, increased in the swCNT group only (Figure 5(d)), and the epithelial cell numbers were only marginally affected (data not shown). swCNT, mwCNT, and ufCBP with OVA significantly increased the macrophage numbers compared to OVA alone (Figure 5(e)), while CNF C was the only CNF sample that significantly increased the macrophage numbers. The percentage of macrophages associated with particles did not differ markedly between the different particle types (Figure 5(f)). However, the CNF D, swCNT, mwCNT, and ufCBP tended to be associated with a lower percentage of macrophages than CNF A, B, and C and were significantly different for swCNT and ufCBP only, as compared to CNF B.

Due to accidental thawing, only half of the BALF samples were analyzed for cytokines, reducing the ability to detect statistically significant differences. The levels of MCP-1 after OVA booster apparently were increased by all particles with OVA except CNF B (Figure 5(g)). The tendencies were most pronounced for CNF A, CNF C, mwCNT, and ufCBP, although statistical significance was reached only for the latter. Although the levels of TNF-*α* were low at this time point (23 days after particle exposure), CNF A and ufCBP with OVA tended to induce higher levels than the other groups (Figure 5(h)).

## 4. Discussion

Studies of airway effects of CNF are scarce in the literature. In the present study, we demonstrate that CNF can promote production of allergen-specific IgE after injection and intranasal exposure to mice. While CNF A and CNF C, and not CNF B and CNF D, demonstrated IgE adjuvant capacity in the injection model, all four CNF samples elicited similar levels of IgE in the airway model. The increase in IgE levels was not associated with a clear allergic airway inflammation in the intranasal model used, since no significant eosinophil influx was observed after allergen booster. Our observation that CNF promote antibody production is in agreement with previous studies reporting allergy-promoting activity of nanosized particles like ultrafine polystyrene particles, ufCBP and CNT [1, 2, 10, 11, 16].

In the intranasal model, the sw and mw CNT samples elicited markedly higher IgE levels than the CNF samples, accompanied with a clear eosinophil airway inflammation. The features of the two CNT samples, making them different from the CNF samples, are the presence of long, thin, and hollow structures (tubes), as opposed to the wider, hat-stacked, more compact structures of the CNF (Figure 1, Table 2). In addition, the present CNT are longer and have 1/2–1/20 of the diameter as compared to the CNF, giving them a higher aspect ratio (i.e., the length/width ratio). Particles with high aspect ratios, such as asbestos fibers, have demonstrated high toxicity, and CNT have been reported to have pathogenic features similar to asbestos fibers [17]. The main characteristic distinguishing nanofibers from nanotubes is the stacking of graphene sheets of varying shapes [18]. While sw and mw CNT consist of single or multiple graphene sheets rolled into concentric cylinders, herringbone (hat-stacked) CNF consist of graphite layers arranged at an angle to the axis of the filament structure, forming a stacked arrangement of cones (Figure 2). This intrinsic difference results in less mechanical strength of CNF compared to CNT [18], as also observed in our study, since the CNF were more susceptible to breaking during the sonication process. In contrast to what has been observed for mwCNT [19], hat-stacked CNF implanted in subcutaneous tissue of rats appeared to become shorter and display decreased degree of aggregation over time [20]. The authors suggested that delamination of graphene layers by hydrophilic substances or energy from cytoplasmic motion may be involved in CNF shortening. Thus, although studies have demonstrated some biodegradation *in vitro *[21], the present and previous data suggest that the CNF samples have lower biopersistence than the CNT samples.

Further, the open, hollow structure of the CNT may be of importance for the allergy-promoting capacity, for instance, as a depot of allergens. Carbon particles have previously been shown to act as allergen carriers [22, 23], and a depot effect has been suggested as one mechanism for particle adjuvant effects [24]. The above mentioned reports support the present finding that tube-associated characteristics, such as a thin, hollow tube structure with assumed high biopersistence, appeared to be particularly important features for the allergy-promoting effect of particles in the airways. CNT also generate reactive oxygen species [25], which probably plays an additional role in their induction of airway allergic inflammation. The capacity of CNF to form ROS is unknown.

Knowledge on which particle characteristics are important for the different biological effects is a prerequisite to enable production of less toxic particles [26]. An advantage of this study was the availability of four CNF batches manufactured in the same facility but with varying particle characteristics deliberately introduced by changing manufacturing conditions [7]. Despite their qualitative differences, the allergy-promoting capacity of the four CNF samples did not differ in the intranasal model, neither with regard to IgE levels nor eosinophil influx. In the footpad injection model, on the other hand, only the CNF A and CNF C increased OVA-specific IgE levels. CNF A and CNF C also tended to differ from the other CNF samples with regard to other endpoints, such as higher levels of IgG1 and IgG2a antibodies in both models and some of the inflammatory markers in the BALF. Although the biological implications of these observations are unclear, they indicate that the CNF A and CNF C samples differ from the CNF B and CNF D samples with regard to induction of biological responses. Common for CNF A and CNF C is a higher metal content, about twice as large relative surface area, a lower mean fiber width, a higher proportion of open channel fibers, and a tendency towards a higher number of defective sites compared to CNF B and CNF D (Table 2). These are all parameters affected by the high temperature treatment performed to reduce the metal content in CNF B and CNF D (described in [7]), and our results therefore suggest that heat-treated CNF may be less toxic with regard to some biological responses. Unfortunately, the association between these heat-treatment sensitive parameters makes it hard to identify single key properties responsible for the higher responses to the CNF A and CNF C. Both metal content, surface area, and structural defects have been suggested to play a role in particle-induced lung inflammation and allergy-promoting effects in mice [16, 27–32]. However, we cannot exclude that the lower fiber mean width and the higher proportion of open channeled fibers in the CNF A and CNF C samples may have contributed to the stronger responses to these particles. Indeed, this would be in agreement with the apparent importance of a thin, hollow structure on the allergic adjuvant effect, as discussed previously.

## 5. Conclusion

The present data demonstrate that CNF and CNT modulate airway responses to allergens, resulting in allergic airway inflammation and production of allergen-specific IgE in mice. When different CNF samples were compared with swCNT and mwCNT, however, the CNT samples appeared to be especially potent in promoting allergic responses, possibly due to their thin, hollow tube structure and assumed high biopersistence. This study provides a basis for studies aiming to further identify the particle properties of importance for airway effects and studies aiming to better understand which airway mechanisms are underlying the allergy-promoting effect of nanoparticles such as CNF and CNT.

## Figures and Tables

**Table 1:** Selected characteristics for CNF A, B, C, and D, swCNT, mwCNT, and ufCBP. The CNF have been thoroughly characterized, reported in Jensen et al. [7]. Data for the swCNT, mwCNT, and ufCBP were previously reported in Nygaard et al. [2].

	CNF A	CNF B	CNF C	CNF D	swCNT	mwCNT	ufCBP, Printex90
Description of morphology (SEM, TEM)^a^							
	Aggregated particles. Medium fraction of fibres, mainly open channel fibres	Aggregated particles. High fraction of fibres, mainly periodically closed channel fibres	Aggregated particles. Low fraction of fibres, mainly open channel fibres	Aggregated particles. Medium fraction of fibres, mainly periodically-closed channel fibres	Aggregated particles. ~50% graphitic material of arbitrary shape ~25% swCNT ~25% mwCNT Open channels	Aggregated particles. High fraction of tubes (>90%), but most are fibres with CNT aspect (the graphite layers make low angles with the channel direction). The fibres contain many defects. Open channels	Aggregated particles. Near spherical particles whose structures are low-graphitized

Percent fibers/tubes versus other carbonous material^a^							
	>50%	>80%	<50%	>50%	~50%	>90%	Not relevant

Fibre/tube widths ± standard error [range] (nm)							
	37.01 ± 1.57 [11.25–108.69]^a^	83.14 ± 4.18 [18.90–302.21]^a^	35.82 ± 2.13 [14.46–185.75]^a^	70.57 ± 2.68 [18.52–286.85]^a^	4.05 ± 0.23 nm [1.41–10.91 nm]^a^	15.04 ± 0.47 nm [7.62–29.01 nm]^a^	
					1.1 nm^b^	20–30 nm^b^	14.3 nm^c^

Fibre lengths							
	Up to 10 *µ*m^a^	Up to 10 *µ*m^a^	Up to 5 *µ*m^a^	Up to 5 *µ*m^a^	0.5–100 *µ*m^b^	0.5–200 *µ*m^b^	Not relevant

Surface area (BET, m^2^/g)							
	103^d^	61^d^	124^d^	56^d^	543^e^	140^e^	321^d^

Impurities							
	>95% carbon^a^	>95% carbon^a^	>95% carbon^a^	>95% carbon^a^	>95% carbon^b^	>95% carbon^b^	>95% carbon^a^
ADF-STEM^a^	High number of Ni [5–500 nm] particles, some large Ti-Fe [>1 *µ*m] particles	Traces of Ni [3–50 nm] and Ni-Fe [50–1000 nm] particles	High number of Ni [10–1000 nm] particles, traces of Cr, Fe and Ti	Traces of Ni [10–50 nm] particles	High number of impurities, mainly Co [1–20 nm], but some Fe [50–500 nm]	Lower number of impurities, mainly Ni-(Fe) [5–40 nm]	Traces
Ni^d^ (AAS, %)	1.29	0.070	4.97	0.036	n.a.	n.a.	<0.0003
Fe^d^ (AAS, %)	0.008	0.023	0.148	0.002	n.a.	n.a.	0.003

Structural defects (*R* = *I*_D_/*I*_G_ as determined by Raman spectroscopy)^d^							
	1.7	0.7	0.9	0.6	n.a.	n.a.	1.2

Endotoxin (ng/mg)^f^							
	<0.0066	<0.0066	<0.0066	<0.0066	0.0079	<0.0066	<0.0066

Acidity (pH)^f^							
	7.34	7.36	7.38	7.35	7.28	7.31	7.32

Abbreviations: ADF-STEM: annular dark field scanning transmission electron microscopy. AAS: atomic absorption spectrometer. BET: Braunauer, Emmet, and Teller method. Fe: iron. Ni: nickel. n.a.: not analyzed. SEM: scanning electron microscopy. TEM: transmission electron microscopy. *R* = *I*_D_/*I*_G_: intensity ratio of the first order bands G and D.

^a^Authors observation by electron microscopy. ^b^Supplied by the manufacturer. ^c^According to Renwick et al. [15]. ^d^Jensen et al. [7]. ^e^Nygaard et al. [2]. ^f^Levels in supernatants after ultracentrifugation of particle suspensions. Endotoxin detection limit = 0.0066 ng/mg (0,25 EU/mL).

**Table 2:** Distribution of selected particle characteristics and the allergy-promoting capacity for all particles tested (as measured by high levels of OVA-specific IgE and eosinophil airway inflammation). To simplify the identification of particle characteristics of importance for the modulated allergy responses after airway exposure, the levels of each particle property or allergic response are subjectively categorized into three levels, illustrated by roman, bold italic, or bold font.

	Main carbon structure	Fraction of fibers/tubes versus disordered material	Fiber/tube width (nm)	Surface area (m^2^/g)	Structural defects (*R* = *I*_D_/*I*_G_)	Metallic contaminants	Allergy-promoting capacity
CNF A	***Fibers, open channels***	***Medium***	***37.01 ± 1.57*** *** [11.25*–*108.69] ***	***103***	**1.7**	**High, mainly Ni**	***Medium***
CNF B	**Fibers, periodically closed channels**	**High**	83.14 ± 4.18 [18.90–302.21]	61	0.7	Traces	***Medium***
CNF C	***Fibers, open channels***	Low	***35.82 ± 2.13*** *** [14.46*–*185.75] ***	***124***	***0.9***	**High, mainly Ni**	***Medium***
CNF D	Fibers, periodically closed channels	***Medium***	70.57 ± 2.68 [18.52–286.85]	56	0.6	Traces	***Medium***
swCNT	**Tubes, open channels**	***Medium***	**4.05 ± 0.23** **[1.41–10.91 nm]**	**543**	n.a.	**High, mainly Co**	**High **
mwCNT	**Tubes, open channels**	**High**	**15.04 ± 0.47** **[7.62–29.01 nm]**	***140***	n.a.	***Less, mainly Ni(Fe)***	**High **
ufCBP	Spherical	Not relevant	Not relevant	**321**	***1.2***	Traces	***Medium***
